# Enhanced Anaerobic Biodegradation and Biomethane Production from Bioplastics by the Addition of Aerobically Prepared Triacylglycerol Lipase

**DOI:** 10.4014/jmb.2504.04047

**Published:** 2025-09-17

**Authors:** Jinok Oh, Jeong Hyeon Hwang, Yebin Han, Gaeun Lim, Sang Ho Lee, Jae-Seok Kim, Shashi Kant Bhatia, Yung-Hun Yang

**Affiliations:** 1 Advanced Materials Program, Department of Biological Engineering, College of Engineering, Konkuk University, Seoul 05029, Republic of Korea; 2 Department of Pharmacy, College of Pharmacy, Jeju National University, Jeju 63243, Republic of Korea; 3 Department of Laboratory Medicine, Kangdong Sacred Heart Hospital, Hallym University College of Medicine, Seoul 05355, Republic of Korea; 4 Institute for Ubiquitous Information Technology and Application, Konkuk University, Seoul 05029, Republic of Korea

**Keywords:** Anaerobic degradation, triacylglycerol lipase, biomethane, polycaprolactone, enzymatic treatment, sludge

## Abstract

This study aimed to overcome the limited biodegradability of bioplastics under anaerobic conditions. With polycaprolactone (PCL) as a model system, the effect of a bioplastic-degrading enzyme, triacylglycerol lipase (TGL), on its degradation and biomethane production was investigated. As the PCL film did not show evidence of any degradation over 14 days under anaerobic conditions in the sludge, TGL from *Bacillus* sp. JY35 was added to promote PCL breakdown into its monomeric form, which could be used for methane production. Application of 200 units/mg of TGL in the sludge led to a 33% increase in PCL degradation over 7 days, with sustained lipase activity despite the decreasing trend in effectiveness after 72 h. Across all type of samples, methane production in the TGL-supplemented sludge increased 1.8-fold across sludge types and up to 2.2-fold when bioplastics other than PCL underwent degradation, compared with that in the untreated sludge. Our result showed the addition of concentrated enzyme could effectively improve bioplastics biodegradability concomitant with methane production under anaerobic conditions, thus offering a feasible approach for optimizing anaerobic degradation with various bioplastics such as Polybuthylene succinate (PBS), and Polybutylene adipate-co-terephthalate (PBAT) although it will take longer time than PCL.

## Introduction

An ideal scenario for bioplastics disposal involved anaerobic environments such as anaerobic digesters, where the breakdown of organic materials in the absence of oxygen offered a viable solution for reducing waste and generating valuable byproducts such as bio-methane [1]. While aerobic degradation of bioplastics has been extensively studied, such was not the case with respect to anaerobic degradation. Typically, anaerobic digesters with sludge showed a slower and less efficient degradation process owing to the limited microbial activity and harsh conditions in such environments [2].

To address this challenge, enzymatic treatment could be an improved method for enhancing anaerobic degradation. Enzymes targeted specific chemical bonds within the polymer structure during bioplastic biodegradation [3]. A range of enzymes, including cellulases, proteases, and lipases, catalyzed the breakdown of different bioplastics [4]. Among these enzymes, lipases were particularly important for the degradation of polyester-based bioplastics such as PCL [5]. Specifically, lipases hydrolyzed ester bonds and broke down polymers into monomers [3] that could be further used by microbial communities. Recently, the use of TGL derived from *Bacillus* sp. JY35 could be utilize for enhancing PCL degradation, as it effectively recognized and cleaved ester bonds in polyesters [6].

Further, TGL obtained from *Bacillus* sp. JY35 has shown potential in breaking down complex polymers such as PCL into its monomeric form, ε-caprolactone, which could be further metabolized by anaerobic microbes [6]. As the activity of the enzyme was not oxygen-dependent, it could function effectively in anaerobic environments. Indeed, although *Bacillus* sp. JY35 was aerobic, its TGL could be used under anaerobic conditions after extraction and enrichment from cell cultures. Thus, by incorporating TGL from JY35 into anaerobic sludge systems, here, we assessed not only its potential for bioplastics degradation but for the ensuing methane production as well, which is a valuable byproduct of anaerobic digestion.

To our knowledge, there were no previous reports of concentrated TGL addition to anaerobic PCL degradation systems; further, most studies have focused primarily on sludge degradation (Table 1). Additionally, several studies have reported that PCL, a commonly used bioplastic, exhibits minimal biodegradability under anaerobic conditions, with degradation percentages as low as 7.6% even after 10 weeks (Table 1), which highlighted the need for improved methods to enhance the anaerobic breakdown of bioplastics such as PCL.

In this study, PCL was used as a model system to test the feasibility of using TGL to improve PCL degradation under anaerobic conditions. The PCL film was added directly to the anaerobic sludge along with concentrated TGL, and methane gas production was analyzed using biomethane potential (BMP) testing. This approach allowed us to assess the biodegradability of PCL and its potential for bioenergy recovery via methane production. By comparing the experimental methane yields with theoretical values, we aimed to quantify the effect of TGL on PCL degradation under anaerobic conditions.

## Materials and Methods

### Chemicals

In all experiments described herein, only analytical-grade chemicals were used. Four types of plastic pellets were obtained from the following sources: PCL from Sigma-Aldrich (USA); Polybutylene adipate-co-terephthalate (PBAT) from SK leveao (Republic of Korea); Polybutylene succinate (PBS) from ANKOR Bioplastics Co. Ltd.,(Republic of Korea); and Polyhydroxybutyrate (PHB) from Goodfellow Cambridge Ltd., (UK). Chloroform was purchased from Junsei Chemical Co. (Japan).

### Sludge Samples

Sludge samples were sourced from the following four sources for biodegradation and methane production analysis: an anaerobic digester sludge in a municipal wastewater treatment plant (Republic of Korea); the water recycling facilities in the Tancheon, Jungnang, and Nanji centers (Republic of Korea), and a brewery wastewater treatment UASB (Upflow anaerobic sludge blanket) reactor (Republic of Korea). Sludge from Bucheon was used in all experiments that are not named. Additionally, three sludges, Sludge 1 from Tancheon, Sludge 2 from Jungnang, and Sludge 3 from Nanji were used to assess their versatility for enhancing methane production. Until the experiment, sludges were refrigerated in an airtight container at 4°C to minimize microbial metabolism and maintain the characteristics of sludge and the microbial community, and immediately before the experiment, it was stored in anaerobic chamber for one to two days to maintain an anaerobic environment. After use in the experiment, it was stored in a freezer at -20°C to preserve the chemical composition of sludge for a long time, and slowly thawed before the experiment to prevent microbial activity from being damaged during the thawing process.

### Preparation of Bioplastic Films

All bioplastic films were prepared using the conventional solvent-casting method [13]. PCL, PBS, PHB and PBAT pellets (0.4 g) were dissolved in 200 ml chloroform, corresponding to a concentration of 0.2% (w/v) pellets in the solvent. The mixture was heated in a water bath at 60°C. After all the bioplastic pellets were dissolved, they were poured into a Petri dish and kept in a fume hood until the solvent evaporated completely. Subsequently, bioplastic films were used in liquid culture.

### Production of Triacylglycerol Lipase

The gene of triacylglycerol lipase (639 bp) was derived from *Bacillus* sp., JY35 in a previous study [6]. The plasmid used for overexpression of triacylglycerol lipase was pET24ma (Kan^r, 1 MCS site with a T7 promoter, lac operator, and p15A ori). *Escherichia coli* strains DH5α and BL21(DE3) were used as hosts for genetic engineering and TGL production, respectively. *E. coli* DH5α (genotype: F^-^ ϕ80lacZΔM15 endA recA hsdR (rk^-^ mk^-^) supE thi gyrA relA Δ(lacZYA-argF)U169) was sourced from laboratory stock, and BL21(DE3) ((genotype: F^-^ ompT hsdSB (rB^-^mB^-^) gal dcm λ(DE3 [lacI lacUV5-T7p07 ind1 sam7 nin5]) [malB^+^] K-12 (λS)) was obtained from Invitrogen [14]. *E. coli* BL21 with pET24ma::TGL culture grown for 24 h was inoculated into LB medium supplemented with 50 μg /ml kanamycin and cultured at 37ºC for 24 h with shaking at 200 rpm [15]. This culture was used to inoculate fresh LB medium supplemented with kanamycin and was maintained under the same conditions (0.1% v/v). After culture absorbance at 600 nm reached 0.6, 0.1 mM isopropyl β-D-1-thiogalactopyranoside (IPTG) was added to induce the production of lipase.

### Ultrafiltration and Enzyme Concentration

The culture solution of the TGL-overexpressing strain was centrifuged to separate the supernatant from the cell pellet. Then, to prevent protein separation and degradation owing to the presence of cells during ultrafiltration, these were removed once more through a 0.22 μM syringe filter (Satorius Minisart). Subsequently, Amicon Ultra-15 centrifugal filters with molecular weight cut-offs of 10 kDa were used to separate cell-induced proteins larger than 10 kDa containing TGL (23 kDa) in the cell-free supernatant. Ultrafiltration was performed at 4°C, 3,008 × g for 40 min, after which, 20 ml of the concentrated supernatant was collected, followed by dividing the sample into two fractions: the concentrate containing the enzyme and the permeate. Protein concentration was determined using the Bradford assay and the protein concentration used in the sludge experiments was 20 to 200 U/mg.

### Anaerobic Degradation with Sludge

Sludge samples (10 ml) were placed in 50 ml serum bottles with a headspace of 40 ml, and 20 mg of bioplastic film was added to each one of them. The serum bottles were closed with rubber stoppers and aluminum caps for all batch fermentation procedures. Nitrogen gas was used to purge the headspace of the serum bottle, thus ensuring that anaerobic conditions were maintained [16]. After cultivation for 1 week, the films in serum bottle were washed several times to remove the sludge, water-soluble monomers. The collected residual PCL films were lyophilized overnight to remove water from their surfaces. All experiments were conducted in duplicate, under shaking at 200 rpm in an incubator kept at 37°C. The weight change of the film before and after degradation, expressed as w/w (weight/weight).

Degradation rate (%) = {Initial film weight (mg) − Remaining film weight (mg)} / Initial film weight (mg) × 100

### GC-FID Analysis

The residual amount of PCL and the degradation yield were determined through GC–FID analysis, prior to which, fatty acid methyl ester derivatization was conducted to prepare the samples [17]. For methanolysis of the samples, a mixture of 1 ml methanol/sulfuric acid (85:15 v/v) and 1 ml chloroform was added, and the vials were heated for 2 h at 100°C. The samples were subsequently cooled to room temperature, and 1 ml of HPLC-grade water was added to the vials, followed by vortexing for 1 min [18]. The organic phase layer at the bottom of the vials was then transferred to a 1.5-ml e-tube containing anhydrous sodium sulfate to eliminate residual water. Samples were filtered (0.2 μm pore size; Chromdisc., Republic of Korea) before injection into the GC–FID equipment. Filtered sample aliquots of 1 μl were then injected into a gas chromatograph (Young-lin 6500, Republic of Korea) operating in split mode (1/10). The chromatograph was equipped with a fused silica capillary column (Agilent HP-FFAP, 30 m × 0.32 mm, i.d. 0.25 μm film) and a flame ionization detector (FID). The inlet temperature was set at 210°C, and helium served as the carrier gas at a flow rate of 3 ml/min. The oven temperature followed a gradient program, starting from 80°C for 0–5 min, and reaching 220°C for 12–17 min. Throughout the experiments, the FID temperature remained constant at 230°C.

### Methane Analysis

The cumulative methane content of the samples was measured by gas chromatography (GC) using a thermal conductivity detector. To produce biomethane, 10 ml Sludge samples were placed in 50 ml serum bottles with a headspace of 40 ml. The serum bottles were closed with rubber stoppers and aluminum caps for all batch fermentation procedures. Nitrogen gas was used to purge the headspace of the serum bottle, thus ensuring that anaerobic conditions were maintained. All experiments were conducted in duplicate, under shaking at 200 rpm in an incubator kept at 37°C. The biogas volume was quantified using a locking glass syringe, followed by withdrawal of the biogas from the headspace. 0.2 ml of biogas were collected from the headspace to confirm the methane content of the total biogas and analyzed using a YL6500 GC (Young In, Chromass) equipped with a 12 ft × 1/8 in. × 2 mm Porapak N-packed column. The oven temperature of the gas chromatograph was maintained at 80°C, and the temperature of the injector and detector of the gas chromatograph were set at 150°C. Methane was quantitatively determined following a method previously described [19].

### Determination of Lipase Activity

Lipase activity was determined spectrophotometrically at 405 nm by measuring the hydrolysis of *p*-nitrophenyl substrates (*p*-nitrophenyl hexanoate, *p*-nitrophenyl octanoate, *p*-nitrophenyl decanoate, and *p*-nitrophenyl dodecanoate). Briefly, 10 μl of the supernatant obtained after removing cells was mixed with 5 μl of *p*-nitrophenyl substrate, 5 μl of ethanol, and 180 μl of 50 mM phosphate buffer (pH 7.4) to make a total of 0.2 ml of mixture for each sample in a 96-well plate. After treatment at 37°C for 30 min, absorbance at 405 nm was measured. According to international parameters, one enzyme unit is defined as the amount of enzyme required to convert 1 μmol of *p*-NPB to *p*-nitrophenol (*p*-NP) per min. All activity assays were performed in duplicate.

## Results and Discussion

### Evaluation of PCL Film Degradation and Enzyme Activity under Aerobic and Anaerobic Conditions

**Fig. 1:**
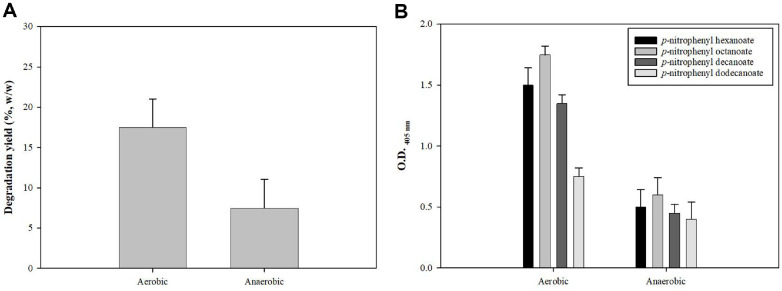
PCL degradation and lipase activity under aerobic and anaerobic conditions. (**A**) Degradation yield after 7 days of degradation under aerobic and anaerobic conditions. (**B**) TGL activity under both conditions using substrates of varying chain lengths (C6, C8, C10, and C12).

Since bioplastics could generally be degraded differently under aerobic conditions than under anaerobic conditions [20], the first experiments were comparison of the extent of PCL degradation in both aerobic conditions and anaerobic conditions. To examine the effect of PCL film degradation under different conditions, we compared the degradation of PCL films by TGL was compared under aerobic and anaerobic conditions, while maintaining the same conditions (37°C, 200 rpm and 7 days) (Fig. 1A). The degradation yield of the PCL film was higher under aerobic conditions (20% weight loss) than under anaerobic conditions (8% weight loss). This finding suggested that the lower degradation rate under anaerobic conditions was due to the lower degradation activity by anaerobic microorganisms, as these inherently had lower degradation capacity than aerobic microorganisms.

Additionally, we measured TGL activity under varying conditions, including different substrate chain lengths (C6, C8, C10, and C12). The reason for starting with C6 was that PCL contains caproate (C6) as the repeating unit [21]. Lipase activity was significantly higher in aerobic than in anaerobic environments across all tested substrate-chain lengths. Although TGL was not an oxygen-dependent enzyme and was expected to show similar activity under anaerobic conditions, these results indicated otherwise. The unexpected reduction in enzyme activity under anaerobic conditions suggested that factors other than oxygen, such as changes in interfacial properties, conformational stability, diffusion limitations, possible inhibitory molecules or other physical and biochemical conditions, may influence enzyme performance [22].

### Effect of PCL Film and ε-Caprolactone on Methane Production

**Fig. 2:**
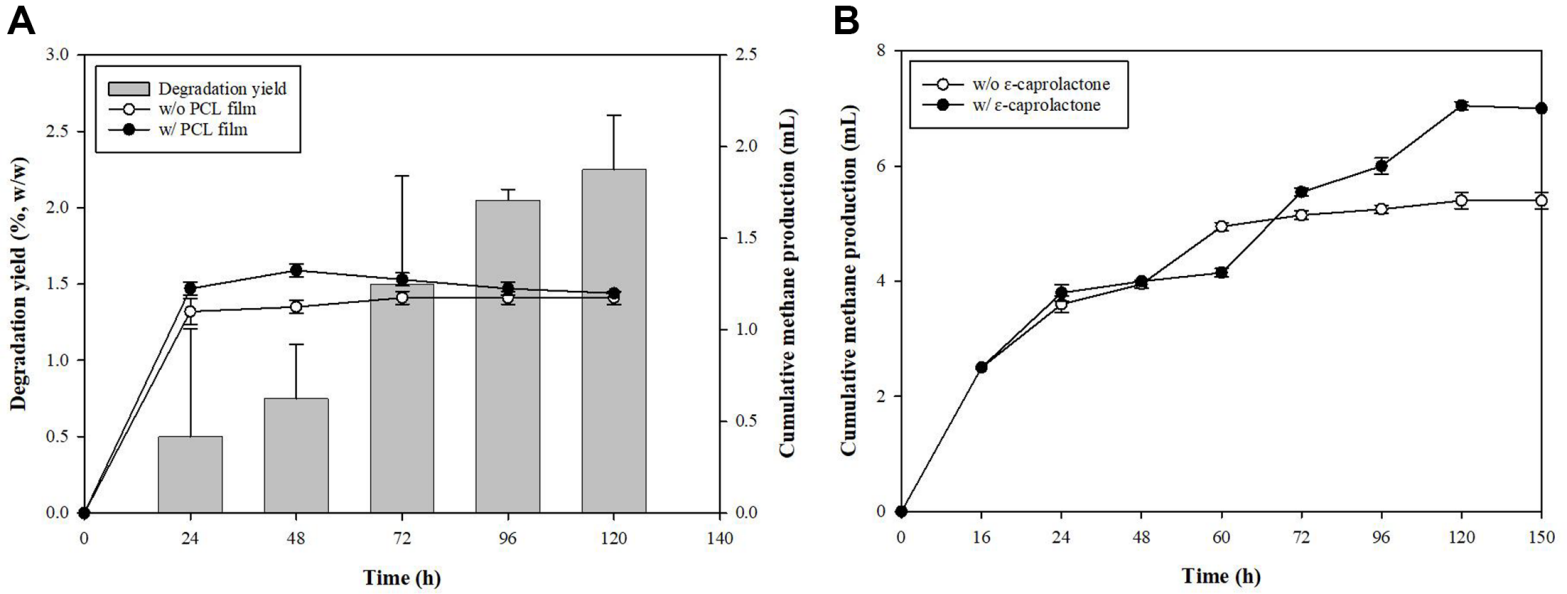
Comparison of methane production by the sludge with or without PCL film and ε-caprolactone over time. (**A**) Biomethane production and degradability tests with or without PCL film. (**B**) Biomethane production with or without ε-caprolactone.

This experiment aimed to determine whether the PCL film might contribute to biodegradation by measuring methane production. To assess this possibility, methane production was compared in the presence and absence of PCL film under in anaerobic sludge. No significant difference was noted in cumulative methane production between the systems with and without the PCL film (control) (Fig. 2A). This finding indicated that the PCL film did not readily degrade under anaerobic conditions; therefore, it seemingly did not contribute to methane production.

Given that the PCL film did not have a significant impact on methane production, we examined whether its monomer, ε-caprolactone, could enhance methane generation. Monomer uptake tests were conducted by adding 16 mM ε-caprolactone to the sludge. Cumulative methane production increased by 12.46% at the end of the experiment, compared to the case without addition of ε-caprolactone), indicating that the sludge could effectively use ε-caprolactone for methane generation (Fig. 2B). The slight but consistent increase in methane production suggested that ε-caprolactone was more easily absorbed and metabolized by microorganisms, leading to enhanced methane production. These findings suggest that for the PCL film to contribute to methane production in the sludge, it must first be broken down to its monomeric form.

### Impact of TGL Concentration on PCL Film Degradation

Previous data in section 3.2 showed that ε-caprolactone, the monomer of PCL, is helpful for methane production, which could only occur with monomer form, not the polymer form. Therefore, the effect of applying only TGL to facilitate PCL breakdown into monomer were tested. Before introducing the experiment, we needed to conduct a control experiment using the well-known protein BSA because of the problem from concentrated protein was functioning as an enzyme rather than merely serving as a nutrient source for the microbial population. Bovine Serum Albumin was added to the experimental setup at the same concentration as that of the enzyme from cultures, and methane production and PCL degradation were monitored over time. The addition of BSA did not result in difference in methane production or PCL film degradation, compared to that without any protein addition (Fig. S1). Additionally, the protein extract before and after filtration was analyzed by SDS-PAGE, as shown in Fig. S2.

To evaluate this, the supernatant from cultures of recombinant *E. coli* expressing TGL, along with a control group (empty vector), was used in degradation tests with a 20-mg PCL film. Initially, the supernatant containing TGL had an enzyme unit of 20 U/mg and showed little degradation, approximately 5%. This finding highlighted the fact that while some degradation occurred, increasing the concentration of TGL was necessary to achieve higher degradation yields. The degradation tests were conducted at varying enzyme concentrations (20, 40, 100, and 200 U/mg).

**Fig. 3:**
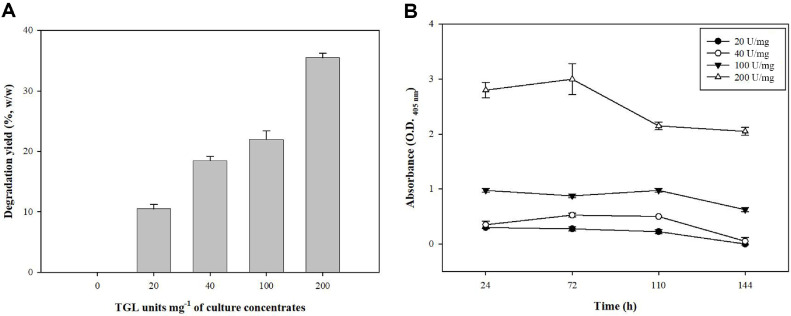
Comparison of PCL degradation with different concentrates of culture filtrates. (**A**) Degradation yield (%) of PCL films and, (**B**) lipase enzyme activity of degradation strains conducted using *p*-nitrophenyl hexanoate (C6).

Increasing the concentration of TGL led to more significant degradation of the PCL films (Fig. 3A). At the highest concentration (200 U/mg), a degradation yield of 32% was observed after 7 days, which demonstrated that higher concentrations of TGL are essential for efficiently breaking down the PCL film. In contrast, lower concentrations (such as 20 and 40 U/mg) yielded much lower degradation rates, underscoring the importance of enzyme concentration in facilitating the degradation process.

In addition to the degradation test, lipase activity was measured using *p*-nitrophenyl hexanoate as the substrate. Fig. 3B illustrates that while lipase activity decreased over time for all enzyme concentrates, the 200 U/mg concentrate maintained the highest activity until the end of the experiment. This suggested that although enzyme activity naturally decreased over time, higher concentrations of TGL allowed for a more sustained activity, which corresponded to the greater extent of PCL degradation observed. These findings lend support to the need for a certain TGL concentration threshold to enhance PCL breakdown into ε-caprolactone.

### Application of TGL Concentrates to Sludge for PCL Degradation

In previous experiments, the application of 200 enzyme units/mg of TGL showed significant effectiveness in enhancing PCL film degradation. Based on these promising results, we applied the same concentration to the sludge to test its potential for improving methane production and PCL degradation under anaerobic conditions. Furthermore, although enzyme activity tended to decrease after 72 h, a 7-day degradation experimental period was established because the emphasis was placed on evaluating the possibility of methanogenesis by PCL degradation.

**Fig. 4:**
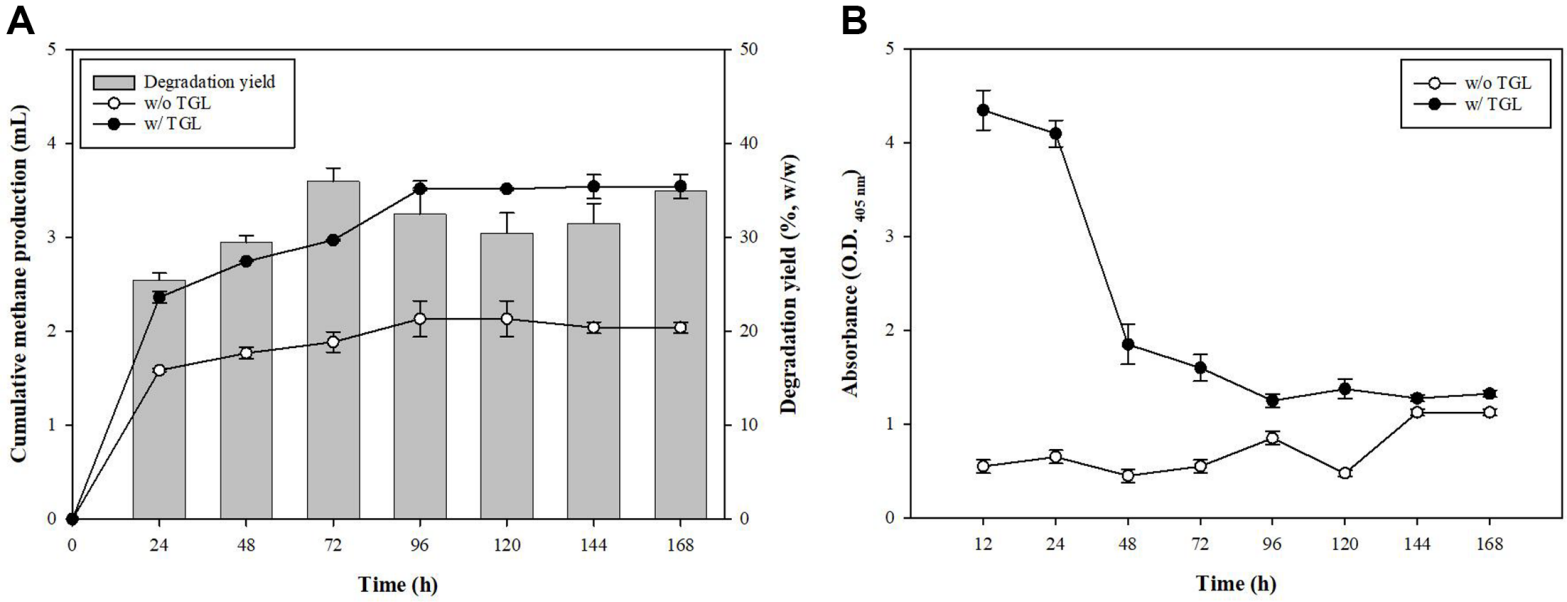
Application of TGL concentrates (200 U/mg) to sludge for PCL degradation. (**A**) Time dependent methane production and biodegradability of PCL films by sludge. (**B**) lipase activity of degrading strains conducted using *p*-nitrophenyl hexanoate (C6).

As shown in Fig. 4A, 10 ml of sludge was mixed with TGL 200 units mg-1 concentrate and then cultured with 20 mg of PCL film. Methane production initially increased but plateaued after 72 h, indicating that no further degradation occurred beyond this point. The total degradability of the PCL film after 7 days was 33%. This result highlighted that while TGL initially promoted methane generation, its effectiveness decreased over time. Further, lipase activity in the sludge was measured using *p*-nitrophenyl hexanoate as the substrate (Fig. 4B). Lipase activity decreased by more than half after 48 h, indicating a decline in enzymatic efficiency. However, lipase activity in the TGL-supplemented sludge remained higher than that in the control throughout the experimental period, suggesting that while enzyme activity decreased over time, the presence of TGL consistently supported higher lipase activity than did sludge without enzyme addition. Further, application of 200 TGL enzyme units in the supernatant to the sludge significantly enhanced the degradation of PCL films and promoted methane production. However, both methane production and enzymatic activity declined after 72 and 48 h, respectively, indicating that the effectiveness of the enzyme decreased over time. Total PCL degradability reached 33% after 7 days. These findings suggested that optimizing the timing and conditions for enzyme application to sustain higher degradation rates and methane yields over longer periods warrants further study.

### Biomethane Production and Degradability in Different Sludges and Bioplastics

**Fig. 5:**
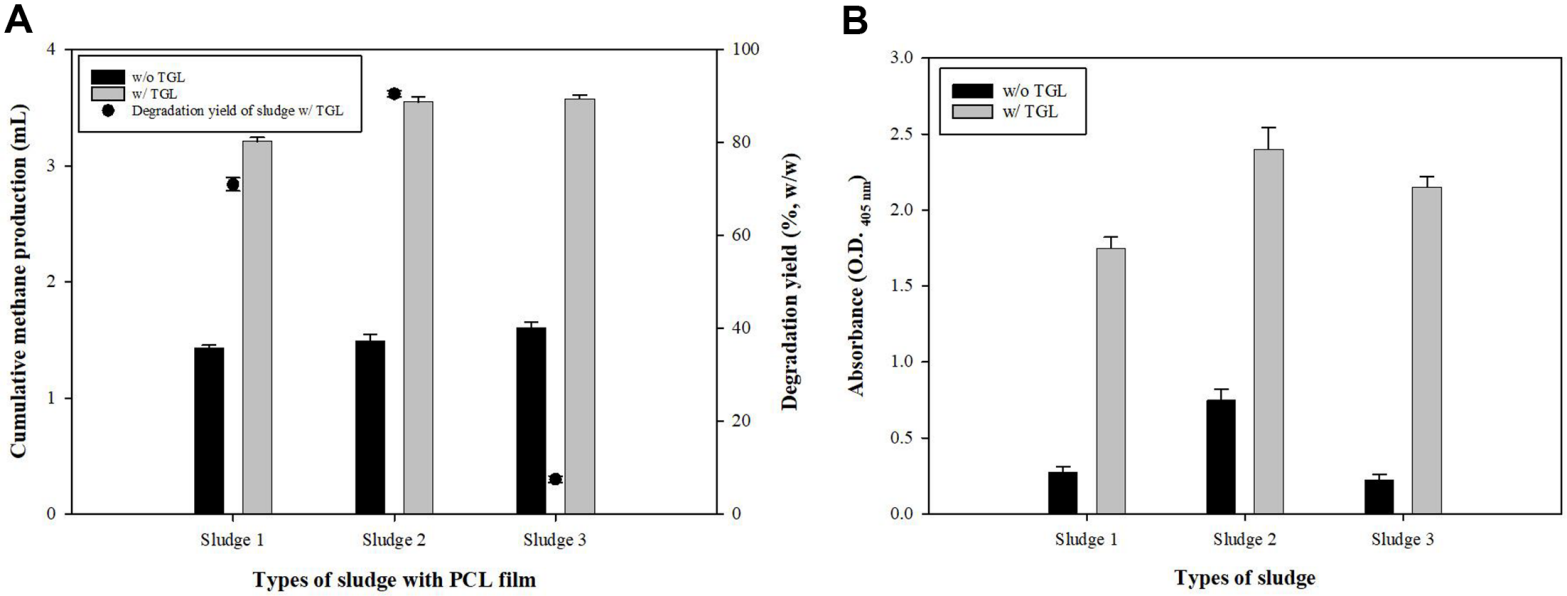
Biomethane production and degradability test using different sludges with or without TGL. (**A**) Biomethane production and degradability between sludge (control) and supplemented sludge in sludge samples of various provenances. (**B**) lipase enzyme activity of degradation strains conducted using *p*-nitrophenyl hexanoate (C6).

**Fig. 6:**
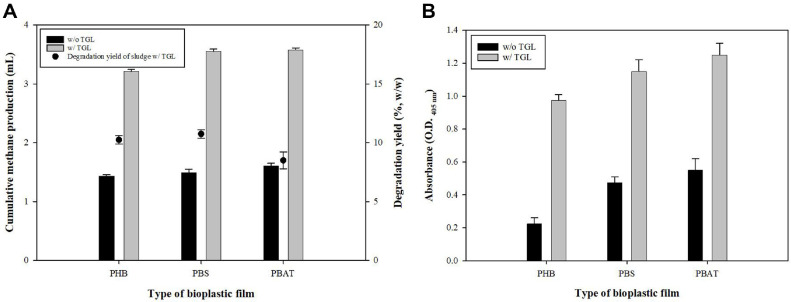
Biomethane production and degradability tests using various types of plastic films with or without TGL. (**A**) Biomethane production and degradability between sludge (control) and PBS, PBAT and PHB plastic film. (**B**) lipase enzyme activity of degrading strains conducted using *p*-nitrophenyl hexanoate (C6).

To evaluate the applicability of TGL for enhancing biomethane production, tests were conducted using various types of sludge and bioplastics. This section assessed the increase in methane production due to TGL addition and evaluated the role of TGL in improving the biodegradability of bioplastics. Methane production of the TGL-supplemented sludge was compared with that of the untreated sludge. Lipase activity was measured using *p*-nitrophenyl hexanoate (C6) as a substrate (Figs. 5 and 6).

Methane production was compared across the three different sludge types under untreated and TGL-supplemented conditions (Fig. 5A). The TGL-supplemented sludge produced substantially more methane, with the highest increase observed in sludge 2. Across all types of sludge, methane production increased 1.8 times, indicating that TGL application generally had a positive effect on biomethane production across all sludge types, regardless of the specific differences among sludges. Fig. 5B further shows that enzyme activity correlated with methane production, with higher activity observed in the TGL-supplemented sludge. These results showed that TGL enhanced lipase activity, which directly contributed to improved biodegradability and methane production.

Similar to PCL, previous studies have found that other bioplastics are also difficult to degrade under anaerobic conditions [23, 24]. Therefore, we checked that the TGL supplementation was effective against other plastics as well, and as a result, it was applied to various bioplastics which have been demonstrated by increased methane yield (Fig. 6A). Similar to the above results, TGL supplemented sludge showed higher lipase activity in all plastics (Fig. 6B). PBS showed the highest methane production, followed by PBAT and PHB. Overall, TGL supplementation significantly improved the biodegradability of PCL and other bioplastics, resulting in methane production in the different sludge types tested.

## Conclusion

This study successfully demonstrated the role of triacylglycerol lipases derived from *Bacillus* sp. JY35 in enhancing anaerobic degradation of PCL. TGL was overexpressed in *E. coli* BL21 with 2 U/mg/ml of enzyme activity in the supernatant. Approximately 20.41 U mg-1 of the enzyme is required per milligram of PCL for effective PCL degradation, which required the concentration of the *E. coli* culture via ultrafiltration. Additionally, the *E. coli* enzyme concentrate required 200 U mg^-1^ of TGL to improve PCL degradation by 33%, while methane production rose 1.8-fold across sludge types. Furthermore, the application of TGL to other bioplastics, such as PBS, PBAT, and PHB, along with different sludge sources, demonstrated even greater potential, with methane production increasing by up to 2.2 times for bioplastics and 1.8 times for different sludges, compared with control conditions.

However, some limitations need to be acknowledged, such as the decline in enzyme activity observed after 72 h and preparation of large quantity of enzymes. This finding indicates that optimizing (*e.g.*, enzyme re-addition, immobilization, pH buffering) the stability and longevity of enzyme activity is crucial for maximizing its effectiveness in continuous anaerobic degradation processes. Future research should focus on enhancing enzyme stability and scaling up the process for industrial applications.

## Supplemental Materials

Supplementary data for this paper are available on-line only at http://jmb.or.kr.



## Figures and Tables

**Table 1:** Previous reports of PCL degradation in sludge under anaerobic conditions.

Type of PCL	Sludge	Temperature (°C)	Period of degradation (days)	Biodegradability (%)	Reference
PCL film	Sludge with mineral salts medium (150 ml)	35	80	ns*	[7]
PCL film	Mineral medium M3 (250 ml) with sludge (10 ml)	35 ± 2	28	ns	[8]
PCL film	100% methane sludge	37	70	7.6	[9]
PCL powder	Sludge (1.4 L)	37	47	92	[10]
PCL-starch blends	Nutrient medium (80 ml) with anaerobic sludge (20 ml)	35	139	83	[11]
PCL powder	Sludge (1.4 L)	55	50	80	[12]
PCL film	Sludge with TGL concentrates (10 ml)	35	7	49	In this study

*Not significant
