# Supplementary material for: Enhanced Anaerobic Biodegradation and Biomethane Production from Bioplastics by the Addition of Aerobically Prepared Triacylglycerol Lipase
Source: J Microbiol Biotechnol. 2025 Sep 17;35:e2504047. doi: 10.4014/jmb.2504.04047 (PMC12463561; doi:10.4014/jmb.2504.04047)
Supplement: Supplementary file 1 [file jmb-35-e2504047-supple.pdf]

J. Microbiol. Biotechnol. 2025. 35: e2504047

https://doi.org/10.4014/jmb.2504.04047

Supplementary Figures

Enhanced anaerobic biodegradation and biomethane production from bioplastics by the addition of aerobically prepared triacylglycerol lipase

Jinok Oh1†, Jeong Hyeon Hwang1†, Yebin Han1, Gaeun Lim1, Sang Ho Lee2, Jae-Seok Kim3, Shashi Kant Bhatia1,4*, and Yung-Hun Yang1,4*

[1]Advanced Materials Program, Department of Biological Engineering, College of Engineering, Konkuk University, Seoul 05029, Republic of Korea

[2]Department of Pharmacy, College of Pharmacy, Jeju National University, Jeju 63243, Republic of Korea

[3]Department of Laboratory Medicine, Kangdong Sacred Heart Hospital, Hallym University College of Medicine, Seoul 05355, Republic of Korea

[4]Institute for Ubiquitous Information Technology and Application, Konkuk University, Seoul 05029, Republic of Korea

+ These authors contributed equally to this work

***Corresponding author**

Prof. Yung-Hun Yang

E-mail address: seokor@konkuk.ac.kr

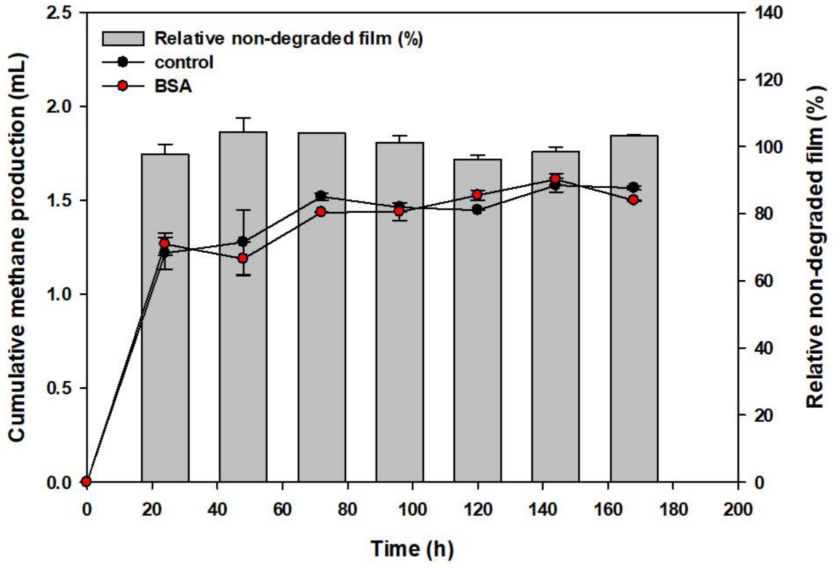

Fig. S1. Time dependent methane production by sludge with bovine serum albumin (BSA).

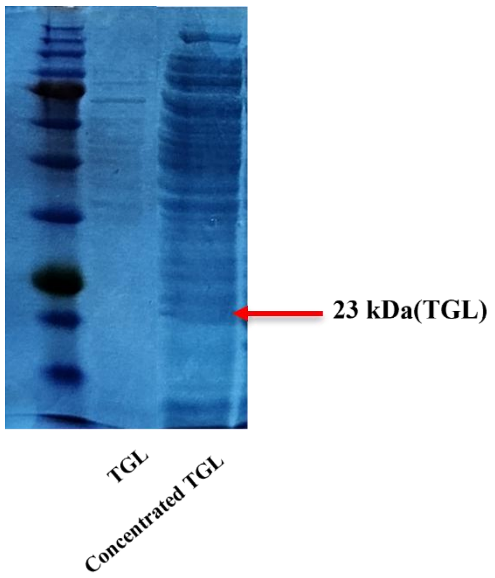

Fig. S2. SDS-PAGE images of protein extracts before (Lane 2) and after (Lane 3) filtration.
